# A Modeling Study for Hip Fracture Rates in Romania

**DOI:** 10.3390/jcm14093162

**Published:** 2025-05-02

**Authors:** Flaviu Moldovan, Liviu Moldovan

**Affiliations:** 1 Orthopedics—Traumatology Department, Faculty of Medicine, George Emil Palade University of Medicine, Pharmacy, Science, and Technology of Targu Mures, 540142 Targu Mures, Romania; 2 Faculty of Engineering and Information Technology, George Emil Palade University of Medicine, Pharmacy, Science, and Technology of Targu Mures, 540142 Targu Mures, Romania; liviu.moldovan@umfst.ro

**Keywords:** hip fracture, orthopedics surgery, osteoporosis, public health, epidemiology

## Abstract

**Background and Objectives**: As the population ages, the number of hip fractures is expected to increase. Developing prevention policies requires knowledge of the factors that lead to the incidence of hip fractures. In this study, we aimed to quantify how much variation in hip fracture incidence can be explained by osteoporosis treatment and temporal trends in the main risk factors. **Methods**: We used the HIP-IMPACT method for a national study in Romania. This method has been validated nationally in Norway, based on the validated IMPACT models for coronary heart disease. The model uses numerical data from 2008 to 2018 on fractures stratified by sex, the prevalence of pharmacological treatments, and risk and prevention factors. **Results**: The HIP-IMPACT model explained 90.1% (4287/4760) of the variation in hip fracture rates recorded during the period of 2008–2018. The increase in the number of fractures was explained by the increase in body mass index (1750/4760; 36.8%), the decrease in the intensity of physical activities (1396/4760; 29.3%), the increase in the prevalence of smoking (1387/4760; 29.1%), the increased prevalence of type 2 diabetes (334/4760; 7.0%), and users of z-drugs (381/4760; 8.0%), glucorticoids (491/4760; 10.3%), and opioids (331/4760; 7.0%). These effects were partially compensated by change over time in the uptake of osteoporosis medication (733/4760; 15.4%), increased prevalence of total hip replacements (491/4760; 10.2%), and reduced benzodiazepine use (559/4760; 11.8%). **Conclusions**: From 2008 to 2018, osteoporosis medication contributed to a decrease in hip fractures by one-eighth, while changes in risk factors and prevention contributed to an increase in hip fractures by three-quarters. There is high potential for fracture prevention through pharmacological treatments but also through national policies to increase physical activity, reduce smoking, and lower body mass index (BMI).

## 1. Introduction

Osteoporotic fractures reflect compromised bone strength and are also known as fragility fractures. They are associated with serious morbidity, disability, reduced quality of life, and excess mortality [1]. Most people who suffer fragility fractures are not diagnosed with underlying metabolic bone disease and are not treated to reduce the incidence of fractures [2]. The most serious is hip fracture, which generates the highest health costs and is responsible for most causes of mortality and morbidity [3].

Along with traditional risk factors for hip fractures, five age-related factors have been identified, including microvascular kidney disease, elevated serum levels of carboxymethyl-lysine, reduced parasympathetic tone, carotid artery atherosclerosis, and elevated levels of trans fatty acids in the blood. Each of these factors is associated with a 10% to 25% increased risk of hip fractures [4]. In Europe, the risk of hip fracture in people over 50 years of age is comparable to the risk of stroke [5].

Although hip fracture rates are decreasing in Europe, with the increase in the population over 60 years of age and life expectancy, new pharmacological treatments and prophylactic measures are needed to maintain this trend [6,7]. To reduce the burden of hip fractures on society and healthcare, an understanding of the factors behind these trends is needed [8]. Reducing hip fracture rates cannot be achieved by prescribing pharmacological treatments for osteoporosis alone [9]. Several studies have suggested lifestyle modification through physical activity, smoking cessation, and decreased body mass index (BMI) [10]. Their qualitative influences are unclear.

As a result of these controversies in the literature, we formulated the following research question: What are the influences of pharmacological treatments and risk factors that contribute to the variation in hip fracture incidence in Romania?

The objective of this study was to quantify the relative contributions of osteoporosis treatment and risk factor changes to the variation in hip fracture incidence in Romania between 2008 and 2018.

## 2. Materials and Methods

### 2.1. Modeling and Data Sources

This modeling study used data taken from the published sources that are cited in this manuscript. The data were processed in accordance with the General Data Protection Regulation and did not require ethical approval.

To explain the changes in the hip fracture rate in Romania between 2008 and 2018, we used the HIP-IMPACT model. This has been published in detail and previously validated in Norway [11]. We adapted the model according to the available data sources and national specifications. The HIP-IMPACT model was developed with the support of the IMPACT model for coronary heart disease [12,13,14]. It has been validated in several countries [15]. In brief, the model aims to be comprehensive, quantifying the contribution of all major risk factors, preventive measures, pharmacological treatments, as well as population attributable risks, to explain the change in hip fracture incidence rate over time [16,17].

Romania differs from Norway in terms of healthcare resource allocation, population health behaviors, disease prevalence, and demographic profiles. To localize the HIP-IMPACT model, national epidemiological data were integrated, including age-specific incidence rates of fractures, population demographics, and treatment uptake rates from Romanian health registries and published studies. When Romanian-specific data were lacking, regional or World Health Organization data for Eastern Europe were used, with appropriate sensitivity analysis, to assess the robustness of the model’s assumptions. Parameters such as risk factor prevalence (e.g., smoking, hypertension, physical inactivity) were adjusted using data from recent national health surveys and validated against known public health trends in Romania. Parameter selection and validation involved internal consistency checks, comparison with independent population-based estimates, and consultation with Romanian clinical experts to ensure the plausibility of outputs in the local clinical and policy context. The HIP-IMPACT model has been validated and has shown high agreement with quality-checked data obtained from medical records in hospitals.

### 2.2. Selection of Variables

We included variables that met the following criteria:

- There is evidence of a causal association with hip fracture (independent (multiple adjusted) RR < 1 or RR > 1), using relative risk estimates for the variable’s association with hip fracture, based on the published scientific literature;
- There are acceptable estimates available to indicate prevalence by sex, in Romania, in 2008 and 2018.

**Figure 1:**
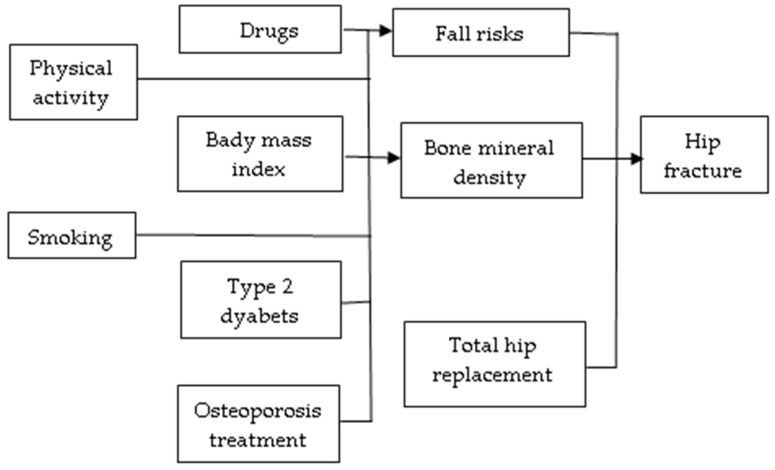
Diagram of causal relationships among the risk factors, the treatments administered, and hip fracture.

For the variables included in this study, causal relationships with hip fractures have been determined through randomized controlled trials and meta-analyses [18]. Figure 1 shows a diagram of the causal relationships among the identified risk factors, the treatments administered, and hip fracture.

The variables included in the HIP-IMPACT model are physical activity, smoking, drugs (glucocorticoids, benzodiazepines, z-drugs, and opioids) affecting bone mineral density (BMD) and fall risk, body mass index (BMI > 25), type 2 diabetes, osteoporosis treatment (alendronate, zoledronic acid, and denosumab), and total hip replacements [19]. Bone mineral density (BMD) was not included in the final model, as it is considered an intermediary of another causal variable that is already included in the model [20]. While BMD is often considered a downstream consequence of both genetic and lifestyle factors, it also serves as an independent and clinically validated predictor of fracture risk. In the causal pathway between treatments/risk factors and hip fracture, BMD is a mediator (Figure 1). The effects of BMD are implicitly modeled by including BMI, osteoporosis medication, glucocorticoid use, smoking, and physical activity. Including BMD in the model would prevent us from quantifying the independent contributions of modifiable upstream factors. However, by omitting BMD, the analysis may underrepresent the full extent of genetic contributions to skeletal fragility, especially since genetic variants can directly influence BMD independent of behavioral or environmental mediators. This exclusion potentially biases the estimation of both direct and indirect effects in the causal pathway. For instance, certain genetic predispositions may increase fracture risk primarily through their impact on BMD rather than through modifiable risk factors, such as diet or physical activity. As a result, this study may underestimate the total genetic effect while overstating the role of lifestyle factors alone. Other variables considered but not included in the model are body height, physical functional level, rheumatoid arthritis, alcohol intake, vitamin D, and some drugs with side effects like diuretics, antihypertensives, hormone therapy, etc.

The prevalence of the risk factors, preventive measures, and treatments followed in the studied period were obtained from reports of the following government institutions: the Ministry of Health, the National Medicines Agency, the National Health Insurance House, the National Endoprosthesis Registry, the National Institute of Statistics, the Competition Council. International sources were also used, including the World Health Organization, World Obesity, Country Economy, etc. In addition, numerous specialized studies published in national and international journals, mostly indexed in PubMed, were used. These are indicated in the Results Section.

When, for the same analyzed variable, we identified several data sources, we preferred the source considered more representative regarding the volume of information provided. For some data that did not partially cover the studied interval, we used regression to extrapolate the prevalence, with the support of the available data. Hip fracture at BMI ≥ 25 was compared with BMI < 25, stratified by sex and three age groups, and the RR with a 95% CI was calculated using a Cox proportional hazards regression model. This was adjusted for age, height, smoking, alcohol consumption, and study region. The association between total hip replacement and the risk of hip fracture was estimated using Cox proportional hazards regression, with total hip replacement considered a time-dependent exposure. For this, residents aged 50 to 80 years without a hip replacement were followed up until hip fracture. They were excluded from follow-up if they had a second hip fracture, left the country, or died. Relative risks, corresponding to 95% confidence intervals (CIs), were extracted from the scientific literature from randomized controlled trials, meta-analyses, or large cohort studies.

### 2.3. Expected and Observed Numbers of Hip Fractures

This study included the entire population of Romania, from 2008 and 2018, which was stratified by sex. Information was collected from reports of the National Institute of Statistics [21]. Information regarding the number of hip fractures in 2008 and 2018 was obtained from nationwide retrospective studies about the incidence and time trend of hip fractures [22,23]. Additionally, primary diagnosis-related group (DRG) data on hip fracture reported by hospitals were collected, with the population stratified by sex (women and men), in 5-year intervals. Information related to primary or secondary diagnosis and surgical procedures for hip fractures was extracted under the following codes: ICD 10 codes S72.0 (femoral neck), S72.1 (trochanteric), and S72.2 (subtrochanteric).

We determined changes in hip fractures rates with the support of a deterministic model that uses epidemiological information between two points in time, delimited by the baseline year (BY) and the end year (EY). The output of the model includes the estimated number of hip fractures requiring explanation (NX^estimated^) and the modeled number of hip fractures explained (NX^modeled^) by the contribution of medication and risk factors.

The estimated number of hip fractures requiring explanation was computed as the difference between the observed number of hip fractures (NO) and the expected number of hip fractures (NE), calculated for the hip fracture rate (r_(BY)_) recorded at the beginning of the study period (BY), as follows:

NX^estimated^ = NO-NE, in the interval (BY-EY), for r_(BY)_ (1)

The modeled number of hip fractures explained was derived from the relationships between the treatments followed and hip fracture risk, as well as between risk factors and hip fracture risks. The model used the best available evidence quantified through meta-analyses and randomized controlled trials. Sex-specific relative risk and risk reduction measures associated with risk factors, preventive measures, and treatments were used. These values were used to determine variations in the proportions of hip fractures corresponding to specific factors, as follows:

(a) Antiosteoporotic treatments applied to patients diagnosed with osteoporosis (NX^med^);
(b) The prevalence of risk factors and preventive measures were calculated based on the variation in the number of hip fractures (NX_i_) corresponding to the temporal evolution of each factor (R_i_), out of a total of n, denoted by NX^risk^.

The two components of the model were summed to explain the variation in fracture count, as presented in the following formula:

NX^modeled^ = NX^med^ + NX^risk^ (2)

### 2.4. Component of the Built Model Based on Antiosteoporotic Treatment

The pharmacological osteoporosis treatment in the model is based on the administration of drugs in the biphosphonate group (alendronate, zoledronic acid, and denosumab). These comprise most fracture-preventive osteoporosis treatments. In Romania, the statistical data regarding the number of users of a particular drug are limited. For this reason, we estimated the number of users of each osteoporosis medication group using statistical data regarding osteoporosis hospitalization episodes [24].

To calculate the explained number of hip fractures (NX^med^), attributable to change over time in the uptake of osteoporosis medication, we multiplied the number of users of each osteoporosis medication (OM) group by the relative risk reduction (∆RR_OM_) associated with the administered treatment and by the hip fracture rate in osteoporosis patients (r_OP_):

NX^med^ = OM × ∆RR_OM_ × r_OP_ (3)

The relative risks were extracted from meta-analyses, randomized controlled trials, or epidemiological studies.

### 2.5. Component of the Built Model Based on Risk Factors

To calculate the number of hip fractures prevented in the end year (EY) from changes over time in the binary risk factor R_i_, the change in the population attributable risk fraction (PARF) was used, as illustrated below:

(4) $${PARF}_{i}=\frac{P_{i}\times ({RR}_{i}-1)}{(P_{i}\times \left({RR}_{i}-1\right)+1}$$

where P_i_ is the prevalence of the risk factor, and RR_i_ is the relative risk of hip fracture associated with presence of the risk factor R_i_.

In cases of harmful exposure, the risk increases, and the PARF can be interpreted as the proportion by which the hip fracture rate would be reduced if the exposure was eliminated. This category includes smoking, type 2 diabetes, the use of medications that increase the risk of falling, etc. In cases of beneficial exposure, the risk is reduced, and the PARF can be interpreted as the proportion by which the hip fracture rate would increase if the exposure was eliminated. This category includes high BMI, high level of physical activity, etc.

The explained number of hip fractures (NX_i_) due to a change in the risk factor prevalence R_i_ was estimated as the product between the expected number of hip fractures in the end year NE_i (EY)_ and the variation in PARF_i_, between BY and EY, as follows:

NX_i_ = NE_i (EY)_ × (PARF_i(BY)_ − PARF_i(EY)_)  (i = 1 − n) (5)

A positive result of the calculation indicates a decrease in the number of fractures due to changes in the respective risk factor levels. A negative result of the calculation indicates an increase in the number of fractures due to changes in the respective risk factor levels. The calculations were made separately based on gender and summed for an overview of the study period.

The risk factors used in the model were collected from cohort studies and meta-analyses based on their individual assessment. But hip fractures occur because of several risk factors that are interrelated. For this reason, the model required an adjustment that assumes the independence of the effects and considers the overlapping prevalence of risk factors.

Therefore, the total number of hip fractures explained by changes in risk factor prevalence (which was obtained by summing the calculations corresponding to each risk factor) were adjusted with the following factor:

(6) $$AF=\frac{CR}{AR}$$

where CR represents the cumulative risk reduction for the “n” specific risks (BMI, smoking, diabetes, etc.), which are denoted by R_i_. It is calculated with the following formula:

(7) $$CR=1-[\prod_{i=1}^{n}\left(1-R_{i}\right)]$$

AR represents the additive risk reduction, which is calculated with the following formula:

(8) $$AR=\sum_{i=1}^{n}R_{i}$$

Therefore,

(9) $${NX}^{risk}={AF\sum_{i=1}^{n}NX}_{i}$$

In Equation (9), a cumulative approach for the risk factors is used, which, unlike the additive approach, avoids repetitive summation of the individual results.

In the next step of modeling, we determined the adequacy of the model [25]. For this, we expressed the difference in percentage between the number of fractures explained by modeling NX^modeled^ and the estimated number of hip fractures that required explanation NX^estimated^. The percentage of hip fractures that was not explained by the model is attributed to the risk factors that were not included in the model, as well as to the uncertainties in the computational estimates.

### 2.6. Data Analysis

Due to the uncertainties surrounding many of the values, we performed multidirectional risk analysis using the extremes analysis method. To this end, we tested the above assumptions. Each parameter of the model was assigned a lower and an upper value. Either 95% confidence intervals were used, where available, or the assigned values plus or minus 20% were used. In this way, we performed extremes analysis, through which the maximum and the minimum feasible values were introduced into the model. Statistical analysis was performed using SPSS–IBM (SPSS, Inc., Chicago, IL, USA) for Windows version 29.0.2 and Excel (Microsoft 365, Albuquerque, NM, USA).

## 3. Results

### 3.1. The Number of Expected Fractures and the Number of Explained Fractures

The population demographics in 2008 and 2018, the corresponding number of fractures, the fracture rates, the expected number of fractures in 2018, and their variation relative to the observed number of fractures in this year, which the model aimed to explain, are presented in Table 1.

In 2008, a total of 11,779 fractures were recorded (of which 63.78% occurred in women and 36.22% occurred in men). In 2018, the number of fractures was 16,732 (of which 68.80% occurred in women and 31.20% occurred in men). With a fracture rate of 6.5/10,000 female inhabitants and 3.87/10,000 male inhabitants recorded in 2008, totals of 7391 fractures in women and 4581 fractures in men were expected in the 2018 population, i.e., a total of 11,972 fractures. But the total number of fractures in 2018 was 16,732. This means that an additional 4760 fractures occurred, (of which 4121 fractures occurred in women and 639 fractures occurred in men), which the model aimed to explain. The HIP-IMPACT model explained 4287 fractures, i.e., 90.1% of the fractures that required explanation (the difference between the expected and observed fracture numbers in 2018). To assess whether this difference was statistically significant, we performed a chi-square goodness-of-fit test, comparing the observed fracture counts in 2018 to the expected values based on the 2008 rates and demographic data. The chi-square test result is χ^2^ (1, N = 22,209) = 392.6, *p* < 0.001. This indicates a highly significant deviation from the expected values, confirming that the observed increase in hip fractures is unlikely to have occurred by chance alone. In addition, we calculated 95% confidence intervals (CIs) for the expected fracture counts using Poisson distribution assumptions, which are appropriate for rare events, like fractures, in a population. As the values observed fall well outside the 95% confidence intervals of the expected values in both groups, we conclude, with strong statistical support, that there was a significant increase in fracture incidence over the decade.

**Figure 2:**
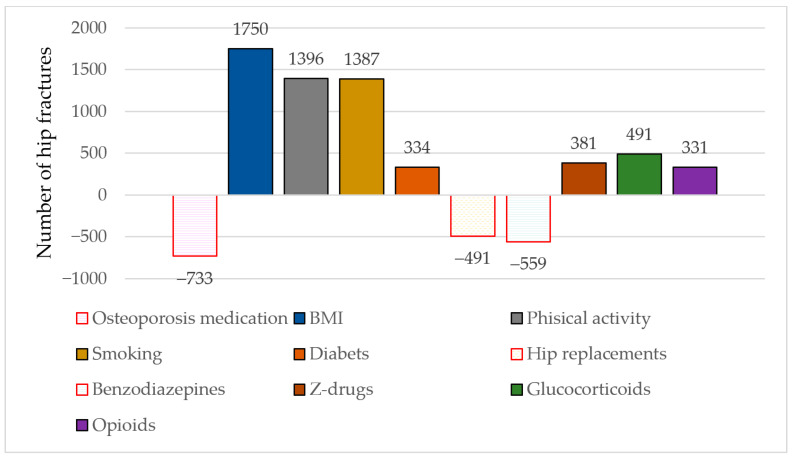
The numbers of hip fractures explained by treatments, risk factors, and preventive measures.

In the sensitivity analysis assumptions, the relative contributions of changes in the specific risk factors and treatment effects remained similar. The minimum and maximum numbers of hip fractures explained by the model were 3189 (67%) and 5902 (124%), respectively. Figure 2 shows the numbers of fractures explained by medical treatments, risk factors, and preventive measures.

While the HIP-IMPACT model explained approximately 90.1% of the variation in hip fractures, our extremes analysis revealed a wide potential range of explanation from 67% to 124%, depending on parameter uncertainty (e.g., relative risks, prevalence estimates). This variability underscores the sensitivity of the model to input assumptions, particularly for risk factors with complex, nonlinear interactions, such as BMI and physical activity. From a policy perspective, this range suggests that while the direction of the model’s conclusions remain valid, the changes in the modifiable risk factors drove most of the observed increase in hip fractures, and the magnitude of each factor’s contribution should be interpreted with caution. Specifically, if the model’s true explanatory power is closer to the lower bound (67%), then unknown or unmeasured variables (e.g., unrecorded medication use, frailty, environmental hazards) may play a larger role than the captured variables. If it trends toward the upper bound (124%), some contributions might be overestimated due to overlapping effects, model structure, or residual confounding. Therefore, policy recommendations should emphasize flexibility and regular data reassessment. Investments in reducing smoking, increasing physical activity, and promoting osteoporosis treatments remain justified but must be monitored for actual impact, ideally supported by real-world effectiveness studies or fall prevention trials. Future iterations of the model should aim to narrow uncertainty through more granular data collection (e.g., stratified by age or comorbidities) and by incorporating lag effects or mediating pathways, such as frailty and fall incidence, which were not explicitly modeled in this version.

### 3.2. Changes in Treatment

From statistical data on hospitalization episodes caused by osteoporosis in Romania [25], we determined the number of episodes reported. This was 8678 patients in 2008, and 3220 patients in 2018, with an average length of hospitalization of 6.24 days. Of these, 75.9% were women, and 24.1% were men.

Table 2 presents the estimated number of total hip fractures explained by changes over time in the uptake of osteoporosis medication, specifically through the administration of drugs in the bisphosphonate group (alendronate, zoledronic acid, and denosumab).

### 3.3. Changes in Risk Factors

The body mass index was obtained from specialized studies [27,28]. One-way sensitivity analysis showed that using the mean BMI would overestimate the effect of BMI, as the correlation between BMI and hip fractures is not linear [29,30]. Therefore, BMI ≥ 25 kg/m^2^ was chosen as the cut-off level. Using World Obesity statistical data, we determined the prevalence of people with BMI ≥ 25 in 2008 (55.2% of women, 61.9% of men) and in 2018 (61.1% of women, 75.1% of men) [31].

The prevalence of self-reported physical activity was obtained from the National Population Health Report [32]. We used the items “resident population according to physical effort when working in a typical week”, “population that rode a bicycle, roller skates, skateboard, etc., for at least 10 min continuously to get to various places and the average number of days of riding a bicycle, roller skates, skateboard, etc.”, as they were collected in both the 2008 and the 2018 surveys. Prevalence data were available for men and women in different age groups. From the Ministry of Health statistics, we deduced the proportion of people who constantly performed physical activities in 2008 (58.1% of women, 54.3% of men) and in 2018 (77.0% of women, 68.2% of men).

According to the Global Adult Tobacco Survey (GATS) 2018, in Romania, 30.7% (5.63 million) of adults aged 15 years and older (40.4% of men, and 21.7% of women) currently use tobacco [33]. Current tobacco use in Romania has increased among all adults (from 26.8% in 2011 to 30.7% in 2018), and among women (from 16.8% in 2011 to 21.7% in 2018) [34].

The prevalence of type 2 diabetes was obtained from the National Population Health Report [32]. The item “chronic diseases in the records of family doctors’ offices in Romania” was used as it was collected in both the 2008 and the 2018 surveys. Prevalence data were available for men and women in different age groups. Using statistical data from the Ministry of Health, we determined the prevalence of people suffering from type 2 diabetes in 2008 (4.2% of women, 6.5% of men), and in 2018 (6.1% of women, 9.8% of men).

From the National Endoprosthesis Registry, we extracted the item “primary hip replacement surgeries”. In 2008, a total of 8974 interventions were performed (5312 women, 3662 men). In 2018, there were 11,949 operations (6886women, 5063 men) [35]. The National Institute of Statistics found that the population of Romania, which, in 2008, was 20.45 million inhabitants (10.49 million women, 9.96 million men), and in 2018, it was 19.47 million inhabitants (9.98 million women, 9.49 million men) [21].

Table 3 presents the estimated number of total hip fractures explained by changes over time in population risk factors and preventive measures.

A total of 4376/4760 (91.9%) fractures were explained by changes in the risk factors and preventive measures. The risk factors that contributed to the increase in the number of fractures were BMI (1750/4760; 36.8%), physical activity (1396/4760; 29.3%), prevalence of smoking (1387/4760; 29.1%), and prevalence of diabetes (334/4760; 7.0%). The factor that decreased the number of fractures was total hip replacement, with (−491/4760; −10.3%).

### 3.4. Changes in Drugs with Side Effects

Statistical data on the use of drugs affecting BMD and fall risk indicate the following population risk factors: for benzodiazepines [36], in 2008, 13.1% of women and 6.4% of men, and in 2018, 6.8% of women and 3.7% of men; for z-drugs (nonbenzodiazepines) [37], in 2008, 21.5% of women and 10.5% of men, and in 2018, 18.00% of women and 9.1% of men; for glucocorticoids [38,39], in 2008, 2.9% of women and 2.1% of men, and in 2018, 4.7% of women and 3.7% of men; and for opioids [40,41], in 2008, 16.4% of women and 13.9% of men, and in 2018, 17.2% of women and 15.00% of men. Z-drugs are a class of non-benzodiazepine hypnotic medications commonly prescribed for the treatment of insomnia. These include zolpidem, zopiclone, and zaleplon. Although pharmacologically distinct from traditional benzodiazepines, z-drugs act on the same GABA-A receptors in the brain and have been associated with increased risk of falls and fractures in older adults due to their sedative effects and potential to impair balance and coordination.

Table 4 presents the estimated number of total hip fractures explained by changes over time in drugs with side effects.

The estimated number of fractures explained by the changes over time in drugs with side effects was 644/4760 (13.5%). The increase in the number of fractures was mainly due to the use of z-drugs (381/4760; 8.0%), glucocorticoids (491/4760; 10.3%), and opioids (331/4760; 7.0%). This situation was partially offset by the increased use of benzodiazepines (559/4760; −11.8%).

## 4. Discussion

To our knowledge, this is the first national study in Romania and the second study worldwide to investigate the effects of changes in treatments and risk factors on the incidence of hip fractures in the population. For this, we used the HIP-IMPACT model, which is an adaptation of the IMPACT model used for coronary heart disease. We found that osteoporosis medication explained about one-eighth, while temporal variation in population risk factors and preventive measures explained about three-quarters of the observed decrease in hip fracture rates. In recent decades, hip fracture rates have increased significantly [22]. With the aging population, a sharp increase in this social burden is anticipated [42]. For these reasons, knowledge of the relative influences of treatment changes and risk factors can limit the burden of future fractures by developing appropriate public health policies and recommendations.

In our study, we had similar findings to those of Kjeldgaard et al. [11] and showed that osteoporosis medication made a modest contribution to fracture prevention. This is explained by the data collected from studies of patients with reported episodes in hospitals, rather than data from real-world settings. The finding that osteoporosis treatments reduced fracture risk by only 15.4% raises important questions about the effectiveness and reach of these interventions in Romania. This modest impact may not reflect intrinsic limitations of the therapies themselves but rather systemic issues within the national healthcare system. Romania continues to experience low osteoporosis treatment coverage, stemming from multiple barriers, including limited access to specialized care, low rates of bone density screening, and restricted availability or reimbursement of medications. Moreover, patient adherence to long-term osteoporosis therapy is often poor, influenced by lack of education, medication side effects, and insufficient follow-up.

The study conducted by Willers et al. [43] in EU countries showed that, despite the high cost of osteoporosis, the use of pharmacological osteoporosis prevention has decreased in recent years. Our results support the need for change in healthcare policies [44]. Therefore, appropriate targeting of osteoporosis medications can reduce fracture risk and thereby reduce healthcare costs. Improving treatments can be supported by fracture liaison services, as Li et al. [45] pointed out. Changes in risk factor levels explain about three-quarters of the variation in hip fractures. The biggest contribution was made by increasing BMI, especially in women. However, Shen et al. [46] found that higher BMI was not significantly associated with the risk of hip fracture in men.

A declining proportion of physically active people in the population explains 29.4% of the increase in hip fracture rates. Physical activity levels are positively associated with hip bone mineral density in a dose–response relationship [47]. Smoking prevalence increased during the study period and explained 29.1% of recorded hip fractures. Smoking is associated with an increased risk of fractures due to decreased weight and BMD, as shown by Yuan et al. [48]. The increase in the prevalence of type 2 diabetes resulted in a 7.0% increase in fracture volume. Even though bone mineral density is often preserved, type 2 diabetes is associated with skeletal fragility, as indicated by Sheu et al. [49]. The 10.2% decline in hip fractures was explained by the increase in the number of people undergoing hip replacement. We found that the decrease in fracture rates was greater in women compared to men because they undergo more interventions [35].

The use of medications with side effects explained 13.5% of the variation in hip fracture rates. Some medications, such as z-drugs, glucocorticoids, and opioids have contributed to the increase in hip fracture rates to a significant extent. Other medications, such as benzodiazepines, have helped decrease hip fracture rates. The negative effects on bones due to glucocorticoids can be counteracted by prioritizing the administration of osteoporosis medications. The prescription of medications that increase the risk of falls has shown variations. There was a decrease in the use of benzodiazepines, which are prescribed to reduce insomnia and anxiety. This explained 11.8% of the decrease in hip fractures [50]. The administration of z-drugs for insomnia contributed to 8% of the increase in the incidence of hip fractures during the studied period [51].

By using the HIP-IMPACT model, we were unable to explain approximately 10% of the variation in hip fracture rates. This is due to some complex variables that could not be included in the model. For example, we did not have a comprehensive model of temporal to account for changes in fall risks. Although reducing the risk of falls through fall prevention programs is effective, their effects on hip fracture rates are inconclusive [52]. Predominantly, these are based on physical activities, which were included in our model. In recent decades, the proportion of elderly Romanians who consume alcohol has increased [53]. However, it is unclear how alcohol consumption affects BMD, fall risks, and hip fracture occurrence. Although nutritional factors and vitamin D supplementation act on bone health, expert reviews do not indicate influences on fracture risks [54].

**Figure 3:**
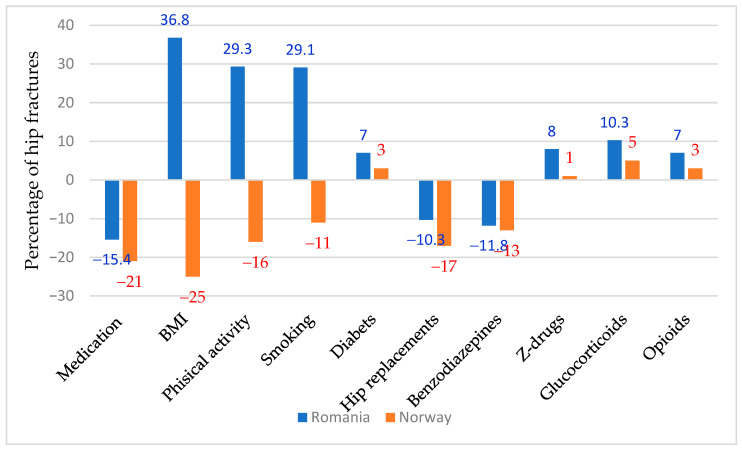
Comparison of the HIP-IMPACT model results in Romania and Norway.

Figure 3 compares the results provided by the HIP-IMPACT model in terms of hip fractures percentages caused by medication, risk factors, and preventive factors, in Romania and Norway. These are the only countries where this model has been applied.

Although the results of our model are generally consistent with the analyses conducted in Norway by Kjeldgaard et al. [11], interesting differences do exist. The contribution from changes in osteoporosis medication was bigger in Norway, exceeding 5%. A significant difference was found in the contributions from changes in population risk factors, including BMI, physical activity, and smoking. In Romania, these factors contributed significantly to the increase in the number of fractures, while in Norway, they decreased the number of fractures This probably reflects the national and regional health insurance policies, which are much more effective in Norway. We also found that the negative effects of drugs with side effects were more pronounced in Romania.

Socioeconomic factors and healthcare policy play a crucial role in shaping population health behaviors and outcomes. Norway benefits from a robust welfare state with high per capita health expenditure, universal access to preventive services, and extensive health education initiatives. In contrast, Romania faces challenges, such as lower healthcare funding, regional disparities in access to care, and limited investment in preventive infrastructure. Economic constraints in Romania may limit access to healthy food, recreational opportunities, and regular medical check-ups, contributing to higher obesity rates and poorer cardiometabolic profiles. Additionally, public health campaigns and school-based health education programs are less systematically implemented compared to Norway. These disparities suggest that individual-level lifestyle interventions in Romania must be supported by systemic policy changes, such as increased funding for preventive care and improved rural health outreach. Understanding these contextual differences is vital for tailoring public health strategies. Efforts to reduce hip fracture risk in Romania must consider structural determinants and cannot rely solely on individual behavior change. Addressing the broader social and policy environment will be essential for meaningful, long-term health improvements.

Studies from Eastern European countries reveal comparable challenges, including underdiagnosis of osteoporosis, low treatment adherence, regional disparities in healthcare access, and a rising prevalence of key fracture risk factors, like obesity and smoking [55]. For instance, Poland has also reported increasing BMI trends and limited uptake of pharmacological osteoporosis treatments despite high fracture rates, pointing to systemic issues that mirror those seen in Romania. Similarly, Bulgaria faces obstacles in providing consistent preventive care and falls short in national coordination for fracture prevention strategies. Kirilova et al. [56] showed that the remaining lifetime probability of a hip fracture in the Romanian population from the age of 50 years is 7.1%, 7.7% in Serbia, 11.2% in Bulgaria, 15.4% in Greece, and 15.9% in Turkey. By situating Romania’s findings within this broader regional landscape, it becomes clear that the modest impact of osteoporosis treatment and the dominant influence of lifestyle-related risk factors are not unique phenomena. Instead, they reflect structural limitations common across many Eastern European healthcare systems, including fragmented care pathways, the underfunding of public health programs, and inadequate implementation of preventive strategies. This reinforces the need for coordinated regional efforts to improve screening, treatment, and public health interventions aimed at reducing the burden of fractures, which is facilitated by studies using the HIP-IMPACT model.

This study has some limitations. The first limitation consists of using data from national registers in Romania, which have a smaller amount of information compared to other countries. Also, some information was not collected based on age groups. Another limitation comes from the limited number and periods of studies regarding the observed variation in hip fracture incidence in Romania. The third limitation comes from relative risk estimates that were not always available across age groups, and sometimes not available for older people. Another limitation stems from the fact that the HIP-IMPACT model does not include all risk factors and possible prevention measures. It does not model the lag times between changes in the risk factor rate and changes in the event rate, which we assumed to be negligible relative to the decade-long interval over which we conducted this study.

Future research directions include updating the model with the best coefficients for reducing the risk of sustaining a hip fracture due to changes in pharmacological treatments and risk factors over time. With the implementation of new national studies over extended study periods regarding the observed variation in hip fracture incidence and the diversification of data from national registries, new, much more detailed research can be conducted. Another research direction is to replicate the model in other countries to determine whether explanatory factors have similar contributions to the variation in hip fracture rates.

## 5. Conclusions

The HIP-IMPACT model applied in Romania to study the variation in hip fractures between 2008 and 2018 showed that one-eighth of the decrease in the number of hip fractures can be explained by the adoption of osteoporosis medications, while three-quarters can be attributed to the reduction of major risk factors. The results of this study suggest that there is a high potential for fracture prevention through pharmacological treatments because of the increase in the proportion of the population over 60 years of age and the increase in life expectancy. National policies that increase physical activity, reduce smoking, and decrease BMI, are also needed.

Our findings underscore the critical opportunity for intervention through targeted behavioral and environmental modifications. To translate these insights into actionable clinical practice, Romanian healthcare providers should integrate comprehensive lifestyle assessments into routine care, particularly in primary and preventive settings. Clinicians must be trained to identify at-risk individuals early and to provide personalized counseling on diet, physical activity, tobacco cessation, and stress reduction. From a public health perspective, national strategies must prioritize scalable and sustainable lifestyle intervention programs. These could include community-based health promotion campaigns tailored to regional cultural and socioeconomic contexts; school and workplace wellness programs to instill healthy behaviors from an early age; subsidized access to nutritionists, fitness programs, and mental health services, particularly in underserved areas; and the integration of digital health technologies, such as mobile apps and telemedicine, to enhance patient engagement and long-term adherence. Moreover, policymakers should align these initiatives with existing national health plans, ensuring that lifestyle intervention becomes a cornerstone of chronic disease prevention. A robust surveillance and evaluation framework is also essential to monitor the effectiveness of these programs and enable continuous improvement.

In conclusion, addressing lifestyle determinants of orthopedic health through coordinated clinical and public health efforts could dramatically reduce the burden of fracture in Romania. This approach represents not only a medical imperative but also a socio-economic opportunity to foster a healthier, more resilient population.

## Figures and Tables

**Table 1:** The expected number of fractures in 2018.

Gender	Population in 2008 [Thousands of Inhabitants]	Observed Number of Hip Fractures in 2008 (NO_(2008)_)	Fracture Rate in 2008 per 10,000 Inhabitants (r_(2008)_)	Population in 2018 [Thousands of Inhabitants]	Observed Number of Hip Fractures in 2018 (NO_(2018)_)	Fracture Rate in 2018 per 10,000 Inhabitants (r_(2018)_)	Expected Number of Fractures in 2018 (NE)	No. of Fractures Requiring Explanation (NX^estimated^)
Women	11,541.5	7513	6.5	11,371	11,512	10.12	7391 95% CI: [7219; 7563]	4121 95% CI: [3949; 4293]
Men	11,000.5	4266	3.87	11,838	5220	4.4	4581 95% CI: [4442; 4720]	639 95% CI: [500; 778]
Total	22,542	11,779	5.22	22,209	16,732	7.53	11,972	4760

**Table 2:** The estimated number of total hip fractures in 2018, explained by changes over time in the uptake of pharmacological osteoporosis treatment of patients with reported hospitalization episodes.

Gender	RR of Hip Fracture per 10,000 People with Osteoporosis ^a^	Fracture Rate in 2008 per 10,000 Inhabitants	Fracture Rate in 2018 per 10,000 Inhabitants	Osteoporosis Hip Fracture Rate per 10,000, in 2008 ^b^	Osteoporosis Hip Fracture Rate per 10,000, in 2018 ^b^	No. of Patients in 2008	No. of Patients in 2018	Relative Risk Reduction ^c^	Number of Fractures Explained (+Generated/−Prevented)	% Explained of Total Hip Fractures
									Best Estimate (Range)	Best Estimate (Range)
Women	6.4	6.5	10.12	41.60	64.77	6586	2443	-	−614 (−722/−521)	−12.9 (−15.1/−10.8)
Men	4.8	3.87	4.4	18.57	21.12	2092	777		−119 (−139/−101)	−2.5 (−2.8/−2.1)
Total						8678	3220	0.53	−733 (−861/−622)	−15.4 (−17.9/−12.9)

^a^ Relative risk stratified by age for all individuals with a value exceeding the threshold for osteoporosis (T-score ≤ 2.5), compared with the risk of the whole population of that age, as reported by Kanis et al. [26]. ^b^ Hip fracture rates in osteoporosis patients were calculated by multiplying the hip fracture rate in the whole population by the RR of hip fracture in osteoporosis patients. ^c^ Table A1 (Appendix A) shows the relative risks used in the HIP-IMPACT model that we considered to be appropriate for the Romanian population, as they were determined from relevant international studies. RRs and 95% confidence intervals (CIs) were obtained from meta-analyses when possible.

**Table 3:** The estimated number of total hip fractures in 2018, explained by changes over time in population risk factors and preventive measures.

Population Risk Factor	Absolute Level of Risk Factor		Change in Risk Factor		Relative Risk	Number of Fractures Explained (+Generated/ −Prevented)	% Explained of Total Hip Fractures
	2008	2018	Absolute Change	Relative Change (%)		Best Estimate (Range)	Best Estimate (Range)
BMI > 25							
Women	55.2	61.1	5.9	10.7	0.66	1028 (872/1209)	21.6 (18.1/25.4)
Men	61.9	75.1	13.2	21.3	0.76	722 (614/848)	15.2 (12.8/17.6)
Total	58.4	67.7	9.3	15.9	–	1750 (1486/2057)	36.8 (30.9/39.8)
Physical activity (%)							
Women	58.1	77.0	18.9	32.5	0.87	964 (821/1134)	20.3 (17.2/23.8)
Men	54.3	68.2	13.9	25.6	0.87	432 (368/508)	9.1 (7.7/10.7)
Total	57.1	73.1	16.0	28.0	–	1396 (1189/1642)	29.4 (24.9/34.5)
Prevalence of smoking (%)							
Women	16.8	21.7	4.9	29.2	1.3	427 (401/501)	9.0 (7.6/10.5)
Men	26.8	40.4	13.6	50.8	1.47	960 (815/1128)	20.1 (17.1/23.6)
Total	19.6	29.1	9.5	48.5	–	1387 (1216/1629)	29.1 (24.7/34.1)
Prevalence of diabetes (%)							
Women	4.2	6.1	1.9	45.2	1.27	162 (134/189)	3.4 (2.8/3.9)
Men	6.5	9.8	3.3	50.8	1.27	172 (144/201)	3.6 (2.9/4.2)
Total	5.2	7.8	2.6	50.0	–	334 (278/390)	7.0 (5.7/8.1)
Total hip replacements (%)							
Women	5.1	6.9	1.8	35.3	0.5	−316 (−371/−268)	−6.6 (−7.4/−5.6)
Men	3.7	5.3	1.6	43.2	0.5	−175 (−204/−147)	−3.6 (−4.2/−2.9)
Total	4.5	6.2	1.7	37.8	–	−491 (−575/−415)	−10.2 (−11.6/−8.5)
Total	–	–	–	–	–	4376	92.0

**Table 4:** The estimated number of total hip fractures in 2018 explained by changes over time in drugs with side effects.

Population Risk Factor	Absolute Level of Risk Factor		Change in Risk Factor		Relative Risk	Number of Fractures Explained (+Generated/−Prevented)	% Explained of Total Hip Fractures
	2008	2018	Absolute Change	Relative Change (%)		Best Estimate (Range)	Best Estimate (Range)
Benzodiazepines							
Women	13.1	6.8	−6.3	−48.1	1.52	−437 (−553/−369)	−9.2 (−10.8/−7.7)
Men	6.4	3.7	−2.7	−42.2	1.52	−122 (−142/−102)	−2.6 (−3.1/−2.2)
Total	10.7	5.7	−5.0	−46.7	–	−559 (−695/−471)	−11.8 (−13.9/−9.9)
Z-drugs							
Women	21.5	18.0	−3.5	−16.3	1.19	295 (249/347)	6.2 (5.3/7.2)
Men	10.5	9.1	−1.4	−13.3	1.19	86 (72/99)	1.8 (1.5/2.1)
Total	16.9	14.3	−2.6	−15.4	–	381 (321/446)	8.0 (6.8/9.3)
Glucocorticoids							
Women	2.9	4.7	1.8	62.1	1.37	316 (268/369)	6.6 (5.6/7.7)
Men	2.1	3.7	1.6	76.2	1.37	175 (148/173)	3.7 (3.1/4.3)
Total	2.6	4.3	1.7	65.3	–	491 (416/542)	10.3 (8.7/12.0)
Opioids							
Women	16.4	17.2	0.8	4.9	1.54	177 (149/208)	3.7 (3.1/4.3)
Men	13.9	15.0	1.1	7.9	1.54	154 (129/181)	3.2 (2.7/3.7)
Total	15.7	16.4	0.7	4.5	–	331 (278/389)	7.0 (5.8/8.0)
Total						644	13.5

## Data Availability

The data used in this study can be requested from the corresponding author.

## Appendix A

**Table A1:** Relative risks used in the Norway HIP-IMPACT model [11].

Treatments	RR (95% CI)
Alendronate, high compliance	0.47 (0.27; 0.81)
Alendronate, medium compliance	0.25 (0.2; 0.3)
Zoledronic acid	0.59 (0.42; 0.83)
Denosumab	0.60 (0.37; 0.97)
Risk/preventive factors	RR (95% CI)
Body mass index ≥ 25 kg/m^2^	Women 50–59: 0.52 (0.40; 0.68) 60–69: 0.66 (0.55; 0.78) 70+: 0.63 (0.57; 0.70) Men 50–59: 0.49 (0.35; 0.68) 60–69: 0.76 (0.62; 0.93) 70+: 0.68 (0.59; 0.77)
Physical activity (>1 h/week)	0.87 (0.8; 0.96)
Smoking	Women: 1.30 (1.16; 1.45) Men: 1.47 (1.28; 1.66)
Type 2 diabetes	1.27 (1.16; 1.39)
Total hip replacements	0.50 (0.48; 0.52)
Drugs with side effects	
Benzodiazepines	1.52 (1.37; 1.68)
Z-drugs	1.90 (1.68; 2.13)
Glucocorticoids	1.37 (1.28; 1.47)
Opioids	1.54 (1.34; 1.77)
